# Linking registries to deliver standardized NCD indicators in the European Health Data Space: Why do we need a Collaborative Health Information European Framework (CHIEF)

**DOI:** 10.3389/fpubh.2025.1684947

**Published:** 2025-11-03

**Authors:** Fabrizio Carinci, Nicholas Nicholson

**Affiliations:** ^1^Departmental Faculty of Medicine, UniCamillus International University of Health and Medical Sciences, Rome, Italy; ^2^European Commission, Joint Research Centre (JRC), Ispra, Italy

**Keywords:** NCD indicators, EU health information systems, federated networks, metadata, diabetes, quality of care and outcomes, European Health Data Space

## Introduction

The availability of actionable information is critical to plan and implement effective strategies for health improvement. Today, the correct governance of health systems requires interpretable models and timely indicators on population needs, quality of care and health outcomes (1).

Despite the increasing availability of health-related data, the capacity of institutions and healthcare organizations to monitor their performance using the available databases, particularly in the area of non-communicable diseases (NCDs), is still hampered by structural factors (2).

In 2015, the Organization for Economic Co-operation and Development (OECD) carried out a global review of health information infrastructure, recognizing the need to overcome critical aspects of access and processing of health-related data (3). Consequently, the OECD released a set of recommendations on health data governance, providing guidance for the secondary use of health data, in compliance with general principles of privacy and data protection (4).

During the last decade, countries including Australia, Finland, France, Germany and the United Kingdom passed new laws allowing the use of health data in the public interest, under specified conditions (5). Such regulations could be used as a platform for deriving indicators that require complex data linkage and analysis.

In the European Union (EU), the implementation of recent regulations such as GDPR (6), the Data Act (7), and the Data Governance Act (8) also had an impact on the ease of linking data between different entities.

These regulations may help removing some of the barriers highlighted by a joint project carried out by the European Commission (EC) and the World Health Organization (WHO). In particular, the final report confirmed that heterogeneous information systems co-exist across Europe, ranging not only in design (from population-based registries to service-oriented databases), but also in information sources (electronic health records, EHRs, to multiple linked datasets) with a varying degree of flexibility, data quality, and sustainability (9).

The report concluded that “the sharing of experiences among neighboring countries or regions may become an important catalyzer.” Other key findings included the heterogeneous information infrastructure at national and sub-national level and relevant methodological challenges arising in the measurement of complex indicators, e.g., multimorbidity and heterogeneous data quality. Furthermore, information on different diseases remained fragmented across different silos and contexts.

In 2025, the OECD confirmed the same difficulties within and beyond Europe, suggesting a three-pronged approach to tackle the critical aspects of heterogeneity, bureaucratic regulations and public trust: (a) definition of common standards and terminology; (b) methods to lower privacy risks and enhance valuable research; and (c) stakeholder engagement (10).

To overcome these problems, a holistic approach may help deal with the diverse operating conditions through which data are processed in different social, political and cultural contexts of European countries, considering disease registries as a key source for NCD indicators (11, 12).

The aim of integrating efforts from different disease areas and multiple disciplines suggests new forms of collaboration to build platforms for the continuous production of indicators across the European Union (EU), a need that will remain a high priority following the implementation of the European Health Data Space–EHDS (13). In 2022, the European Commission started the “Healthier together” programme to identify and implement effective policies and actions to reduce the burden of major NCDs (14). The 5-year initiative is expected to roll out a stream of activities to enhance the response of health systems to a range of conditions, including health determinants, cardiovascular diseases, diabetes, chronic respiratory diseases, mental health, and neurological disorders. The scale of the challenge in NCDs requires overcoming the stated barriers to data processing and analysis through a common overarching approach.

In support of this programme, the Commission's Directorate-General for Health and Food Safety (DG SANTE) launched an initiative coordinated by the Joint Research Center (JRC) to deliver the essential elements of a common EU indicator framework that could be applied seamlessly across major NCDs. The “collaborative health information European framework” (CHIEF) is an EU think- tank of new ideas and solutions, specifically designed to overcome the difficulties and hindrances toward the regular collection of NCD indicators experienced by recent EU projects.

The scope of CHIEF is “to provide expert input in the form of concepts and solutions for the design and implementation of a sustainable information system that will enable the periodic collection and progressive scalability of an EU-harmonized set of indicators across NCDs, within the current and future flagship initiatives.”

Although focusing on the indicator-collection process, the aim of CHIEF is to tackle the broader perspectives of the interoperability of systems and the advanced statistical and epidemiological approaches needed to assess and integrate the study of comorbidity into the analysis of NCDs.

The initiative has the merit to consider expectations of the public as an integral part of the plan, establishing the necessary interrelations across fundamental flagship programmes such as the joint action on cardiovascular diseases and diabetes (15) and the EHDS.

Important issues concerning data quality, metadata representation, the application of valid statistical methods and data cleaning/harmonization are also considered as part of the CHIEF initiative.

## Structure of the collaboration

To expedite the realization of its goals, the initiative has been conceived as an agile forum, through which experts can collaborate within and across disease groups, toward the finalization of activities that will be run through a mix of remote and in-person work, teleconferences and annual meetings.

The work of experts has been coordinated through the formation of multidisciplinary “design working groups,” each tackling the separate challenges involved within three different points of focus that are consistent with the three-pronged strategy proposed by the OECD (10):

- *“Metadata,” to define the type and contents of the documentation required to integrate the different data sources available for the continuous production of NCD indicators across different disease domains*. This point of focus includes challenges regarding the information infrastructure, including *common data elements* (CDEs) with specific attention for risk factors across NCDs, *evaluation methods* (reliability of data sources for the calculation of indicators, data quality score and capture-recapture methods) and *target indicators* (epidemiology, prevention, diagnosis, treatment and outcomes). In this context, a key interest is the FAIR-ification of data elements used for the production of indicators, i.e., making indicators “Findable, Accessible, Interoperable, and Reusable” (16).
- *“Federated data analysis,” showcasing relevant analytical solutions that can help the EU and Member States (MS) to report on NCDs effectively, by resolving the major methodological challenges of sharing information from the existing data sources, without sharing personal data*. This point of focus addresses a series of methodological challenges, including those related to the longitudinal analysis of federated databases using specific parameters (e.g., time to diagnosis), and how to process heterogeneous databases avoiding bias and misinterpretation (17).
- *“Barriers and enablers toward implementation*,” *understanding the barriers hampering the implementation of regulations, with the direct participation of relevant stakeholders*. This point of focus addresses challenges regarding the social barriers to the implementation of NCD information systems, including the effects of current EU regulations and the role of stakeholders such as decision makers, health professionals and people with NCDs. The specific angle of the direct participation of citizens in the calculation of indicators has been considered as a neglected aspect that will be redressed within CHIEF.

CHIEF has taken diabetes as its starting point due to the inroads already made in a stream of projects carried out by the European EUBIROD network (18), through its identification of the key elements of the points of focus specified above for the progressive expansion toward additional disease domains (19–23).

## Methodology

The methods adopted to operationalise each point of focus are consistent with the theory of learning health systems (LHS) on three different levels (24):

- “Practice to data,” i.e., how the data are collected in real life situations, including the following tasks:

a) *Indicator framework, diabetes indicators core set*, involving a multidisciplinary review of the key literature on the different purposes and scope of NCD indicators, focusing on those considered most relevant for governance and planning.
b) *Metadata: definition, semantic description, and FAIR-ification*, to conceive a generic model for describing the indicators, including the intrinsic quality of constituent data elements and how semantic linkage can be achieved to make them interoperable with other metadata, using FAIR data principles.

- “Data to knowledge,” i.e., how data is analyzed to understand phenomena, including:

a) *Federated software to measure the impact of risk factors and data quality*, to present the details of the federated method applied to validate cardiovascular risk prediction in diabetes for the guidelines of the European Society of Cardiology (25), using cohorts extracted from selected diabetes registers in Europe;
b) *Federated analytics to monitor the impact of risk factors in NCDs*, to compare the accuracy of different CVD prediction models in diabetes through the previously specified federated method;
c) *Federated data analysis framework*, to revise the state of the art in the application of federated methods and compared their suitability, with practical examples of how NCD indicators could be effectively derived;
d) *Automated review of data sources and registers across NCDs in EU countries*, using a mixed set of qualitative and quantitative methods and tools to routinely update the contents and quality of data sources, taking CVD risk stratification in diabetes as an exemplary;
e) *Operational plan to build a coherent EU information system for NCDs*, to design a set of targeted studies that can use the specified methods to support the implementation of NCD indicators in the EHDS according to CHIEF;

- “Knowledge to practice,” i.e., how indicators are used by policy makers, doctors and patients to improve outcomes, including the tasks:

a) *Privacy, data governance and ethics in the handling of sensitive data*, applying legal and data governance expertise to compile principles of relevant EU regulations with best practices in data governance, interoperability and ethics. All components were embedded into a validated instrument that can be routinely used to benchmark the level of compliance of data controllers and data holders.
b) *Stakeholders engagement*, in which clinical experts and patients' representatives cooperated to revise methods and tools to measure the barriers, attitudes and roles of people with diabetes toward the routine data collection and sharing of health information.

The methods were initially discussed in plenary sessions and later assigned to specific “clusters,” according to the specific background of participating experts.

## Results

The results obtained from the application of these methods will be presented in detail in different papers, corresponding to each of the tasks specified above in the points of focus.

All papers were extracted from a series of reports delivered by the diabetes design working group (“CHIEF-diabetes.dwg”), composed primarily of experts from the EUBIROD network.

The reports revised best practices and delivered methods and tools:

1) to specify purpose and scope of selected NCD indicators;
2) to contextualize indicators and their common data elements (CDEs) by adopting a model that can highlight strengths and weaknesses of the results obtained with varying level of detail, taking data quality and standardization into due account;
3) to compute standardized indicators using a feasible and secure method for federated analytics;
4) to benchmark compliance of data controllers and data holders against common standards of privacy, data governance and ethics;
5) to evaluate the views and expectations of patients toward data collection and the routine use of health indicators, as a key strategy to enhance public trust;

All technical reports contribute to the calculation of a set of standardized indicators in diabetes, as a basis for the implementation of a platform specifically designed to produce NCD indicators on a rolling basis.

## Conclusions

The first stream of results obtained by CHIEF will help define the foundations of a common health information system that can act as a model for the continuous monitoring of NCDs.

In CHIEF, the direct participation of citizens has been acknowledged as a key determinant for the delivery of high-quality indicators that matter to people with NCDs. Such recognition represents a characteristic aspect of the proposed approach, whose goal is to close the information loop between “data to knowledge” and “knowledge to practice” in LHS (24).

According to this model, collecting new evidence using real-world data does not guarantee by itself that data incorporating new knowledge will be available in everyday practice. To reach this goal, a multilateral collaboration is required between different types of stakeholders, including clinicians, analysts, policy makers and citizens. Accurate information must be available to all counterparts to improve transparency and interpretation with minimal bias.

For each focus, CHIEF takes into full consideration the “pillars” represented by the set of social, scientific, technological and cultural aspects at the basis of EU policies and regulations (such as the GDPR, EHDS, Data Act, Data Governance Act, etc.).

The framework considers the current systems of data collection applied by institutions such as EUROSTAT, OECD, WHO, International Diabetes Federation (IDF), and global standards applicable by data sources and registers networks such as the International Consortium for Health Outcomes Measurement (ICHOM).

The outputs of CHIEF can be used as the building blocks of a modern form of LHS that can accelerate the uptake of indicators using data and digitalisation to connect clinicians and patients for the common goal of sustainable health improvement.

The deliverables have been finalized to ensure that indicators considered by CHIEF are not only evidence based and policy oriented, but are also feasible and achievable through small pilot projects that can be used as a proof of concept of the functionality of the framework.

To support European policies, CHIEF seeks to provide a sustainable and practical solution for a periodic collection of NCD indicators that has so far proved elusive. The activity contributes to the resolution of critical aspects addressed internationally by the OECD, in an EU context that despite its complex integration strategies and many relevant projects, still presents critical barriers to the effective use of health information.

Recognizing the inherent complexities, CHIEF keeps the focus at the broader generic level of NCDs, rather than on specific challenges within any one disease domain. In its initial specifications, diabetes has been taken only as an exemplary case of more complex situations, applicable to all chronic diseases and multimorbid conditions.

The initial phase of CHIEF will close at the end of year 2026, when a final report will discuss the main results obtained, outlining the key recommendations for the following steps of the European Commission, toward the regular publication of fully contextualized NCD indicators.

These results will help predefine the elements needed by the EHDS to operate a system of unbiased indicators, considering all the relevant functions activated in a federated system of data catalogs with searchable metadata functionalities (26). They also come at a timely moment to support the EU4Health programme (27), while also addressing the three strategic directions of the World Health Assembly's 2023–2030 NCD implementation roadmap (28).

The collaborative experience of registry networks may provide unique insight into the clinical content and epidemiological interpretation of health information accessible to the EHDS, making both CHIEF and the EHDS complementary to each other and able to provide the right synergies for meaningful comparisons across the EU.
